# Direct evaluation of the antioxidant properties of salivary proline-rich proteins

**DOI:** 10.3164/jcbn.19-75

**Published:** 2020-06-09

**Authors:** Tomoko Komatsu, Kyo Kobayashi, Yoshinari Morimoto, Eva Helmerhorst, Frank Oppenheim, Masaichi Chang-il Lee

**Affiliations:** 1 Division of Dentistry for the Special Patient, Department of Critical Care Medicine and Dentistry, Kanagawa Dental University Graduate School of Dental Medicine, 82 Inaoka-cho, Yokosuka, Kanagawa 238-8580, Japan; 2 Yokosuka-Shonan Disaster Oral Health Research Center & Oxidative Stress/ESR Laboratories, Kanagawa Dental University Graduate School of Dental Medicine, 82 Inaoka-cho, Yokosuka, Kanagawa 238-8580, Japan; 3 Department of Critical Care Medicine and Dentistry, Kanagawa Dental University Graduate School of Dental Medicine, 82 Inaoka-cho, Yokosuka, Kanagawa 238-8580, Japan; 4 Department of Molecular and Cell Biology, Boston University Henry M. Goldman School of Dental Medicine, Albany street, Boston, MA 02118, USA

**Keywords:** saliva, proline-rich proteins, oxidative stress, reactive oxygen species, antioxidant

## Abstract

Proline-rich proteins are associated with the formation of an acquired protein layer overlying the tooth enamel surface. Previous studies have described the antioxidant activity of salivary histatin against the hydroxyl radical from Fenton’s reaction, acting as the critical reactive oxygen species. However, the role of proline-rich proteins in mitigating the oxidative stress caused by reactive oxygen species in the oral cavity remains unclear. In this study, we investigated the antioxidant effects of proline-rich proteins 2 on direct reactive oxygen species using electron spin resonance spectroscopy. For the first time, we demonstrated that proline-rich proteins 2 exhibits antioxidant activity directly against the hydroxyl radical produced by hydrogen peroxide with ultraviolet. Considering that identical results were obtained when assaying 30 residues of proline-rich proteins 2, the direct antioxidant effects against the hydroxyl radical by proline-rich proteins 2 may be related to these specific 30 residues.

## Introduction

Oxidative stress due to the activity of reactive oxygen species (ROS) such as the hydroxyl radical (HO^•^) or superoxide (O_2_^•−^), and antioxidant imbalances, is related to various lifestyle-related diseases including hypertension, arteriosclerosis, diabetes, myocardial infarction, cerebral infarction, and cancer.^(1–3)^ However, antioxidant systems comprising antioxidant enzymes and antioxidants, play a protective role by removing ROS.^(4–6)^ To maintain homeostasis of the oral cavity, an adaptive balance is sustained between oxidative stress caused by ROS and the antioxidant system. An imbalance in this environment due to an increase in oxidative stress augments the risk of systemic diseases including various oral diseases.^(7,8)^ We have previously reported that a failure to balance antioxidant activity with oxidative stress levels can further exacerbate diabetes mellitus, a disease that is linked to the oral disease periodontitis.^(9–11)^ Excess oxidative stress in the synovial fluid was also observed in human and animal models of temporomandibular disorders.^(12)^ In addition, ROS release has been reported to cause a decrease in the antioxidant capacity of saliva, leading to the development of oral cancer in smokers.^(13,14)^ Interestingly, the antioxidant activity of salivary vitamins has been reported to be effective against the oxidative stress caused by the oral lichen, planus.^(15)^

Studies have established that antioxidant activity, including that of the saliva and salivary proteins, is crucial in maintaining homeostasis, influencing the progression of systemic diseases, and sustaining the overall health of the oral cavity.^(16,17)^ Salivary proteins are reported to be involved in homeostatic processes, lubrication, antibacterial activity, and control of tooth demineralization and remineralization.^(18)^ These proteins are comprised of a mixture of protein and peptide derived from the salivary glands, oral exudates, and cell debris, and have a protective effect in the oral cavity.^(19–23)^ Thirty percent of these mixtures are made up of small proteins called saliva-derived peptides.^(24,25)^ The four major saliva-derived peptides are histatin, cystatin, statherin, and proline-rich proteins. It is well established that saliva contains high concentrations of proline-rich proteins (PRPs), secreted from the salivary glands.^(26–29)^ PRPs have been reported to play a role in the formation of the acquired enamel pellicle, a thin film that is formed by the selective binding of salivary proteins onto the enamel surface of the teeth. Therefore, PRPs function as a part of this protective protein layer that covers the tooth surface, with acidic PRPs having a high-affinity binding site in their phosphorylated domain for hydroxyapatite.^(18)^ PRPs interact with several oral bacteria, can inhibit the formation of hydroxyapatite, and suppresses crystal growth of calcium phosphate, by binding calcium ions from a supersaturated solution of hydroxyapatite, thereby protecting the tooth enamel.

Studies have found that measuring the levels of the antioxidant protein SOD^(30,31)^ or relative lipid peroxidation^(32)^ of salivary proteins are effective tests to assess oxidative stress in the oral cavity. However, there are only a few reports on the antioxidant activity of saliva, and specifically salivary proteins. Salivary PRPs are characterized by high amounts of the amino acids proline, glycine, glutamine, and glutamic acid, and make up over 60% of total salivary peptides.^(16,33)^ These proteins are typically grouped into the following categories: acidic (aPRP), basic (bPRP), and glycosylated (gPRP).^(16)^ We recently confirmed that histatin could reduce ROS-induced oxidative stress, especially that caused by HO^•^ (via Fenton’s reaction), using electron spin resonance (ESR) spectroscopy.^(34)^ However, there are few, if any studies that assess the direct effects of PRPs on ROS generation. Therefore, in this study, we investigated the effect of PRPs, especially PRP2, on the generation of ROS, using ESR. These findings provide novel evidence characterizing the antioxidant properties of salivary PRPs.

## Materials and Methods

### Reagents

Reagents were purchased or sourced from the following: xanthine (X) and xanthine oxidase (XO) (grade III) were obtained from a chromatographically purified suspension of buttermilk in 2.3 M (NH_4_)_2_SO_4_ and 10 mM sodium phosphate buffer (pH 7.8) containing 1 mM EDTA and 1 mM sodium salicylate, respectively. Superoxide dismutase was obtained from Sigma (St. Louis, MO), hydrogen peroxide (H_2_O_2_) and FeSO_4_ were purchased from Wako Chemical (Osaka, Japan), and 5,5-dimethyl-1-pyrroline-*N*-Oxide (DMPO) was soured from Labotech (Tokyo, Japan).

Synthetic PRP2 was obtained from the American Peptide Company (Sunnyvale, CA) and from Quality Controlled Biochemicals (Hopkinton, MA). Human parotid secretion (HPS) was collected from five healthy volunteers aged 25 to 38 years. Informed consent was obtained and approved by all participants, according to the protocol from the Institutional Review Board at Boston University Medical Center. The HPS was collected using a Curby cup device that fits into the opening of the Stensen duct. The flow of HPS and salivary secretions stimulated by sour candy were collected in a graduated cylinder on ice. A 25 µl aliquot of parotid secretion (PS) was seeded on blood agar (Hardy Diagnostics), to confirm that the collected PS was sterile and free of whole saliva contamination. The PS protein was dialyzed and lyophilized, dissolved in buffer A [50 mM Tris-HCl and 50 mM NaCl (pH 8)], and then eluted using a MonoQ HR16/10 column (Amersham Biosciences, Uppsala, Sweden). In addition, a gradient step (using buffer B containing 50 mM Tris-HCl buffer and 1 M NaCl (pH 8) at a rate of 2 ml/min; time distribution: 0–38 min, 0–13% buffer B; 38–233 min, 13–22% of buffer B; 233–250 min, 22–40% buffer B) was performed. The purity of the synthesized PRP2 was confirmed using polyacrylamide gel electrophoresis to assess the separation of the positive and negative ions in the compound, followed by reverse phase chromatography.^(35)^

### Determination of protein concentrations

The protein concentration of each sample was measured using the micro-bicinchoninic acid (BCA) protein assay kit (Pierce Chemical, Co., Rockford, IL), with bovine serum albumin as the protein standard.

Electron Spin Resonance technique. HO^•^ was produced via Fenton’s reaction (H_2_O_2_/FeSO_4_) as reported previously.^(5,6)^ Briefly, a reaction mixture of H_2_O_2_ (20 µM) and FeSO_4_ (20 µM), in 0.1 M phosphate-buffered saline (pH 7.2) containing 50 mM DMPO as the spin trap, with or without pretreatment by PRP2 or 30rPRP, was used. The mixture was transferred to a flat cell, and the DMPO-OH spin adduct was measured using the X-band ESR spin-trap method. For the HO^•^ generated through the ultraviolet (UV)/H_2_O_2_ reaction system, a reaction mixture containing 10 mM DMPO and 20 mM in H_2_O_2_ in 0.1 M phosphate-buffered saline (pH 7.2), with or without pretreatment by PRP2 or 30rPRP, was used. The mixture was transferred to a flat cell and irradiated using a PAN UV lamp at 365 nm and 40 mW. After 20 s of UV irradiation, the DMPO-OH spin adducts were measured using the X-band ESR spin-trap method.^(5,6)^

O_2_^•−^ was generated via the X/XO reaction system, as previously reported.^(5,6,36)^ O_2_^•−^ was generated from xanthine oxidase (0.1 U/ml) and xanthine (362 µM) in 0.1 M phosphate-buffered saline (pH 7.2), containing 50 mM DMPO, with or without pretreatment by PRP2 or 30rPRP. The mixture was transferred to a flat cell, and the DMPO-OOH spin adduct was measured using the X-band ESR spin-trap method.

All ESR assessments were conducted using the JES-RE 3X, X-band spectrometer (JEOL, Tokyo, Japan) instrument. The ESR device was connected to the WIN-RAD ESR data analyzer (Radical Research, Tokyo, Japan), with the following settings: microwave output, 8.00 mW; magnetic field, 334.8 ± 5 mT; field modulation width, 0.079 mT; receiver gain, 400; sweep time, 1 min, and time constant, 0.03 s. The hyperfine coupling constant was calculated using the resonant frequency measured with the microwave frequency counter, and the resonant field measured by the JEOL ES-FC5 field measurement unit. The detected spin adducts were quantified from the ESR spectrum of a manganese oxide standard. The actual measured signal intensity was expressed as relative height, normalized to the signal intensity of the ESR spectrum of a manganese oxide standard.^(5,6)^ All experiments were repeated independently, at least four times.

### Statistical analysis

Statistical analysis using Dunnett’s test (OMS, Saitama, Japan) showed that the results from the experiments conducted in this study were normally distributed. Experimental results are expressed as mean ± SD. The relative means from the 3 or 4 different protein concentration levels were compared using a two-way analysis of variance. *P* values less than 0.05 were considered statistically significant.

## Results

### Effects of PRP2 on HO^•^ generation by the Fenton’s reaction system

**Fig. 1:**
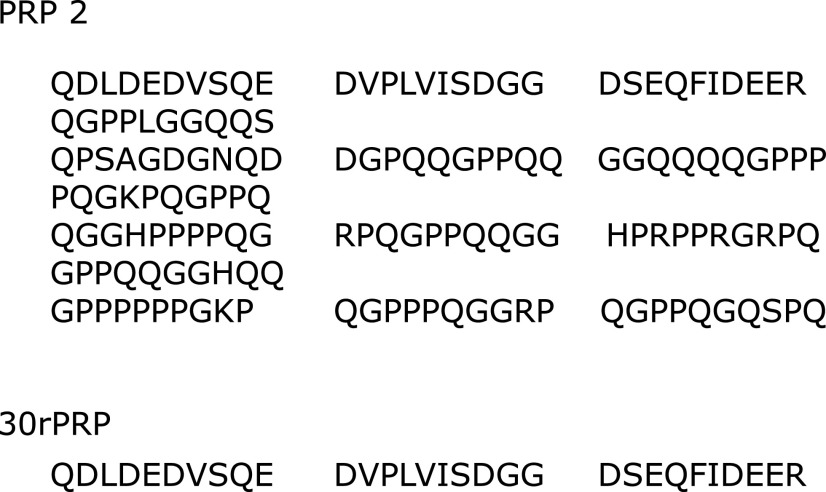
The amino acid sequence of proline-rich protein 2 (PRP2) and 30rPRP.

The amino acid sequence of the salivary protein PRP2 used in this study is shown in Fig. 1. The only basic residue in this peptide is arginine, and there are no aromatic residues present. Conversely, the other major salivary protein, histatin 5, consists of 24 residues, of which none are proline, with 14 basic residues and 3 aromatic residues. This could indicate that histatin displays different antioxidant properties when compared to PRP2 or other PRPs.

**Fig. 2:**
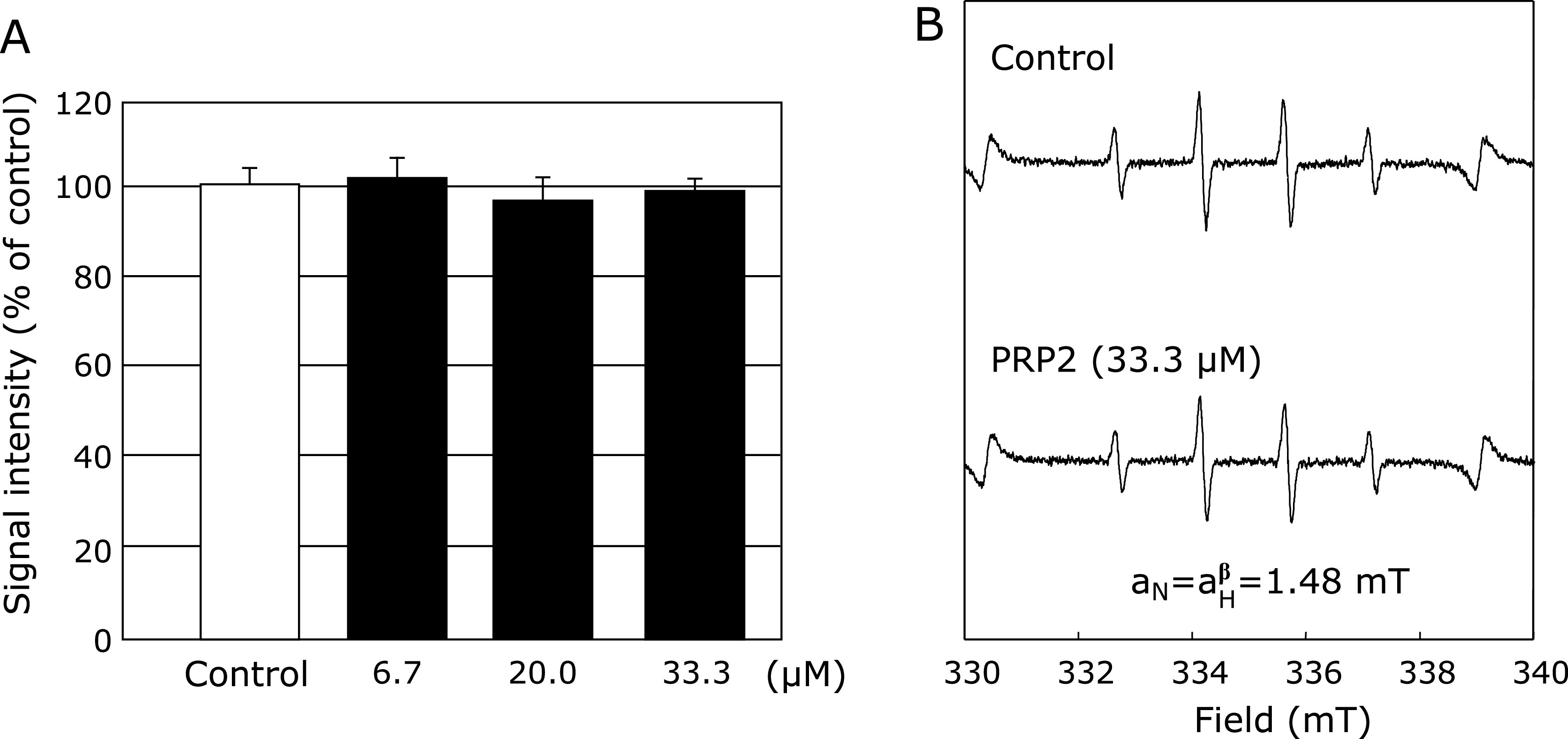
Effects of different concentrations of PRP2 (6.7, 20.0, and 33.3 µM) on HO^•^ generated by the Fenton reaction. (A) Dose-response of PRP2 or the control on HO^•^ generation from H_2_O_2_ and FeSO_4_. (B) ESR spin trapping measurement of HO^•^ generated from H_2_O_2_ and FeSO_4_ in 0.1 M PBS, using 50 mM DMPO as the spin trap in the absence of PRP2 (control), or under conditions of PRP2 (33.3 µM), respectively.

The effect of PRP2 on HO^•^ produced from Fenton’s reaction was examined by ESR spin trapping, using DMPO as the spring trap. As previously reported,^(5,6)^ after adding H_2_O_2_ to FeSO_4_, a characteristic DMPO-OH spin adduct spectrum was observed, with hyperfine splitting resulting in four typical peaks. However, in the reaction with FeSO_4_ and PRP2 (6.7, 20, and 33.3 µM) pretreatment at different concentrations, and the subsequent addition of H_2_O_2_, the DMPO-OH signal did not change at each concentration of PRP2 (Fig. 2).

### Effects of PRP2 on HO^•^ generation by ultraviolet irradiation of H_2_O_2_

**Fig. 3:**
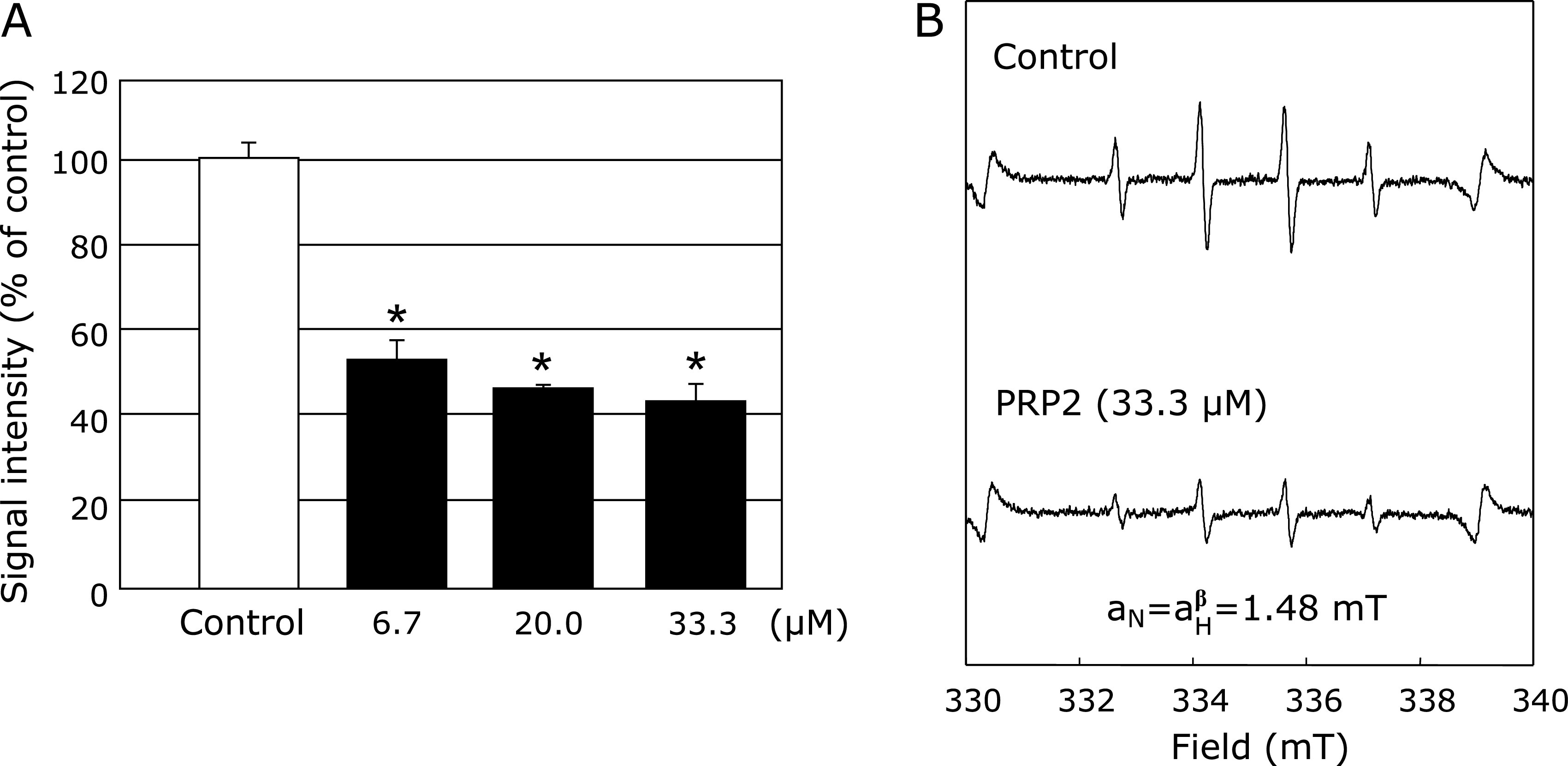
Effects of different concentrations of PRP2 (6.7, 20.0, and 33.3 µM) or the control on HO^•^ generated by ultraviolet (UV)/H_2_O_2_ reaction system. (A) The dose-response of PRP2 and the control on HO^•^ generated by UV irradiation with H_2_O_2_ is represented. (B) ESR spin trapping measurement of HO^•^ generated from UV irradiation with H_2_O_2_ in 0.1 M PBS, using 50 mM DMPO as the spin trap, in the absence of PRP2 (control), or under conditions of PRP2 (33.3 µM) pretreatment, respectively. *Significantly different (*p*<0.05) from the corresponding control value.

The effects of PRP2 on HO^•^ generated using the UV/H_2_O_2_ reaction system were examined by ESR spin trapping, using DMPO as the spin trap. As reported previously,^(5,6)^ after the UV/H_2_O_2_ reaction occurred, a characteristic DMPO-OH spin adduct spectrum was observed, with hyperfine splitting resulting in four typical peaks. When H_2_O_2_ was pretreated with different concentrations of PRP2 (6.7, 20 and 33.3 µM) followed by UV irradiation, it was apparent that the DMPO-OH signal had significantly decreased as compared to the control (Fig. 3).

### Effects of PRP 2 on O_2_^•−^ generation

**Fig. 4:**
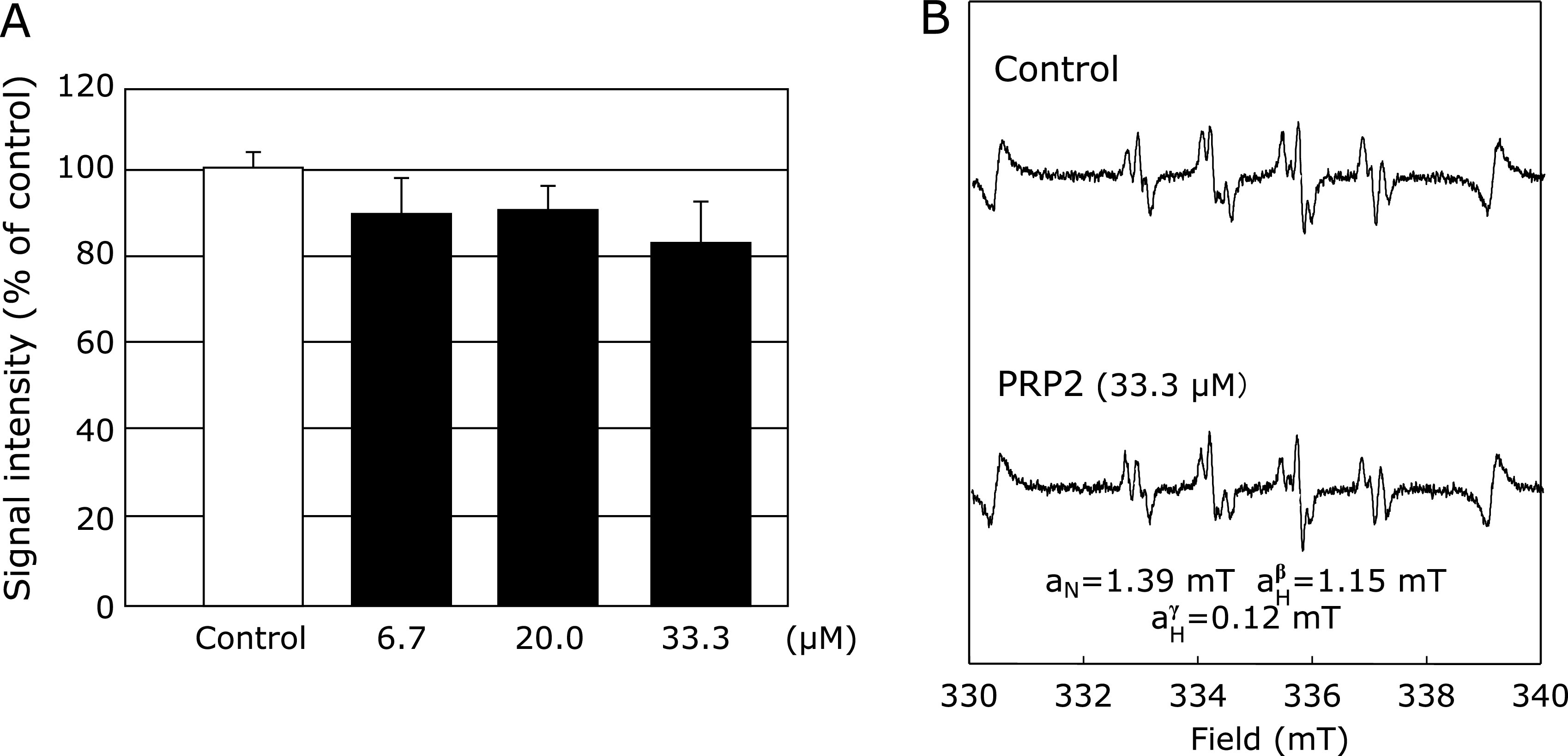
Effects of different concentrations of PRP2 (6.7, 20.0, and 33.3 µM) on O_2_^•−^ generation from xanthine (X)/xanthine oxidase (XO) reaction system. (A) Dose-response of PRP2 or the control on O_2_^•−^ generation from X and XO. (B) ESR spin trapping measurement of O_2_^•−^ generated from XO (0.1 U/ml) and X (362 mM) in 0.1 M PBS, using 440 mM DMPO as the spin trap, in the absence of PRP2 (control), or under conditions of PRP2 (33.3 µM) pretreatment, respectively. *Significantly different (*p*<0.05) from the corresponding control value.

As reported previously,^(5,6)^ following the X/XO reaction, a characteristic DMPO-OOH adduct spectrum was observed, with hyperfine splitting giving rise to 12 resolved peaks. These signals were quenched by 150 U/ml superoxide dismutase, thus confirming that they were derived from O_2_^•−^ (data not shown). However, with XO and PRP 2 (6.7, 20 and 33.3 µM) pretreatment at different concentrations, and the subsequent addition of xanthine, the DMPO-OH signal did not change at each concentration of PRP2 (Fig. 4).

### Effects of 30rPRP on ROS generation

**Fig. 5:**
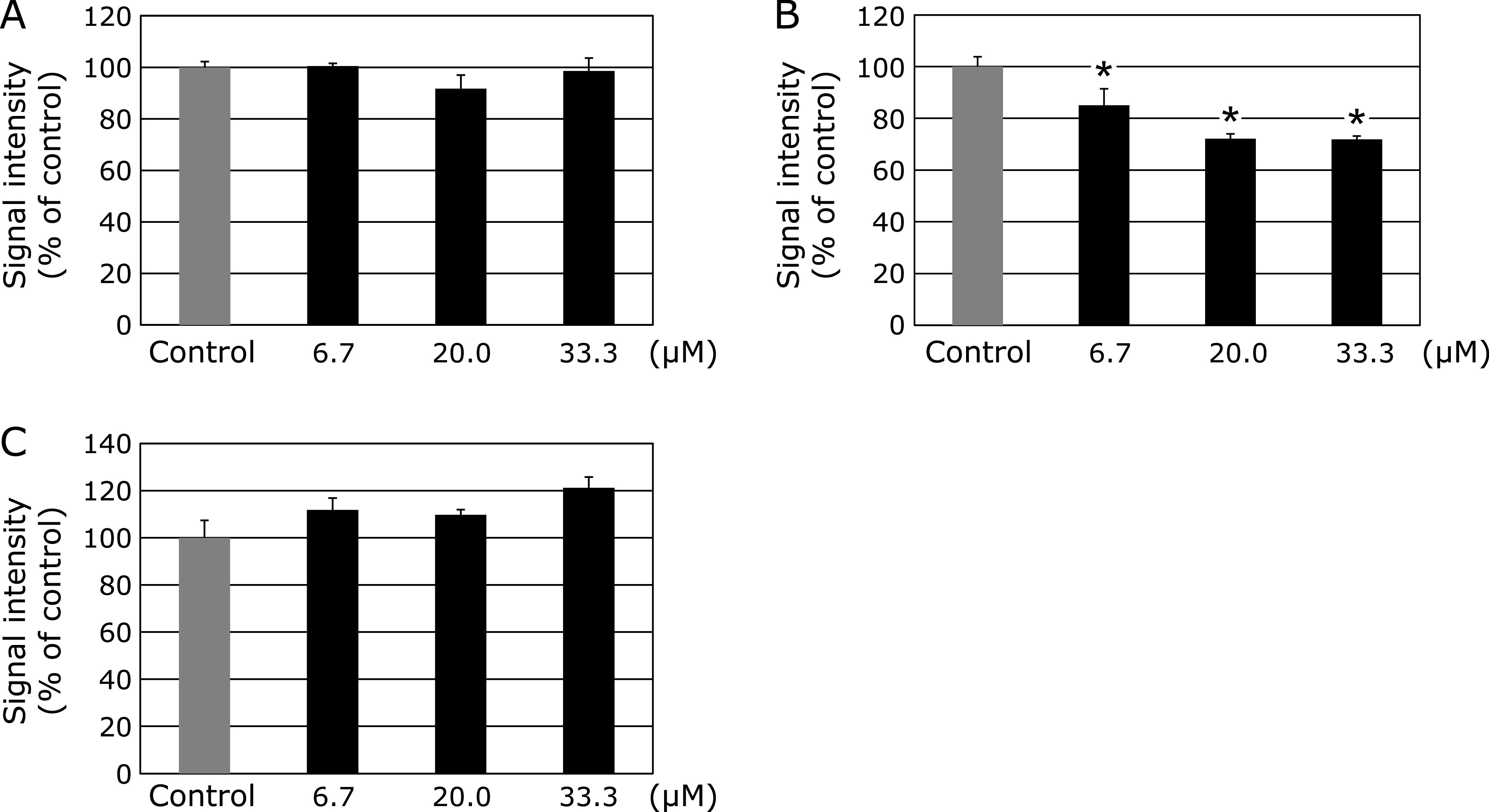
Effects of 30rPRP (6.7, 20.0, and 33.3 µM) on ROS generation. (A) Dose-response of 30rPRP or the control on HO^•^ generated from the Fenton reaction. (B) The dose-response of 30rPRP and the control on HO^•^ generated from the UV/H_2_O_2_ reaction system is represented. (C) Dose-response of 30rPRP and the control on O_2_^•−^ generated from the X/XO reaction system. *Significantly different (*p*<0.05) from the corresponding control value.

Finally, to establish and clarify the correlation between the structure of PRP2 and antioxidant activity, we investigated the antioxidant activity of 30rPRP, a 30-residue structure contained in PRP2. With different concentrations of 30rPRP (6.7, 20 and 33.3 µM) pretreatment of H_2_O_2_ from Fenton’s reaction (FeSO_4_/H_2_O_2_) system, it was apparent that the DMPO-OH signal did not show any change at each concentration of 30rPRP (6.7, 20 and 33.3 µM) (Fig. 5A). When H_2_O_2_ was pretreated with different concentrations of 30rPRP, followed by UV irradiation, we observed that the DMPO-OH signal was reduced (Fig. 5B). When O_2_^•−^ was generated from the X/XO reaction system with 30rPRP pretreatment at different concentrations (6.7, 20 and 33.3 µM), the DMPO-OOH signal did not change at any concentration of 30rPRP (Fig. 5C).

## Discussion

PRPs are comprised of approximately 25–40% of the amino acid proline and are the main salivary proteins produced by the parotid and submandibular glands, accounting for almost 70% of total protein in human saliva.^(29)^ PRPs have a phosphorylated domain that has a high affinity for hydroxyapatite. These proteins play an important role in the formation of enamel film, which functions as a protein covering on the surface of the tooth.^(18,37)^ It has been shown that salivary peptides such as histatin show antibacterial activity.^(38)^ We have recently reported the direct antioxidant activity of histatin using the ESR method, suggesting that salivary proteins display both antioxidant activity and oral antibacterial activity.^(34)^ However, previous studies have not investigated the actual effect of PRPs on the generation of ROS. Therefore, we used ESR to examine the direct antioxidant effects of PRPs, especially PRP2, on the production of ROS such as HO^•^ and O_2_^•−^. This study provides the first evidence that PRP2 directly suppresses HO^•^ production, though it did not reduce the level of O_2_^•−^ generation from the X-XO reaction system (Fig. 1–4).

We studied the effects of PRP2 on HO^•^ generation in both Fenton’s reaction system and the UV/H_2_O_2_ reaction system. PRP2 did not affect HO^•^ generation by Fenton’s reaction (Fig. 1), but significantly suppressed HO^•^ generation from the UV/H_2_O_2_ system (Fig. 2). Therefore, this suggested that PRP2 may directly eliminate HO^•^ without iron chelation. We have already confirmed that histatin suppresses the production of HO^•^ generated by Fenton’s reaction system, without affecting HO^•^ generated from the UV/H_2_O_2_ reaction system.^(34)^ It is interesting to note that the same salivary protein had different effects on HO^•^ production^(34)^ (Fig. 2 and 3). In addition to Fenton’s reaction, the generation of highly reactive HO^•^ *in vivo* is important in a variety of ROS-induced diseases, including oral diseases.^(12,39,40)^ These findings suggest that PRPs and other salivary proteins such as histatin, are coupled, and have an antioxidant effect on HO^•^. Consequently, this might be important evidence for the physiological defense capabilities of salivary proteins in the oral cavity.

In general, homeostatic balance is maintained by ensuring equilibrium in the generation and removal of ROS in biological systems. The breakdown of this balance due to increased production of ROS in the oral cavity is known to lead to an augmented risk of oral disease. ^(7,8)^ Additionally, we have reported that oxidative stress caused by this disruption increased the risk of specific oral diseases such as periodontitis, and systemic diseases such as diabetes and cardiovascular diseases. ^(9,39)^ In periodontal diseases, ROS-induced oxidative stress is associated with a chronic inflammatory response caused by bacteria, resulting in alveolar bone resorption. ^(9,11)^ In fact, neutrophils from the peripheral blood of patients with acute apical periodontitis (AAP) have been reported to increase ROS production, particularly in response to treatment of chronic periapical granulomas. ^(41,42)^ Furthermore, antioxidant vitamins that exhibit antioxidant activity are known to be effective against oral diseases such as the oral lichen planus ^(15)^. It was confirmed that PRP2 directly suppressed the generation of HO^•^ (Fig. 3). These results are the first direct evidence of the antioxidant effects of PRPs on ROS. Previously, we conducted similar antioxidant research tests on all PRPs (PRP1–4). The reason PRP2 was chosen from all PRPs for this study was that the antioxidant effects of HO^•^ by the Cu ion used in Fenton’s reaction showed different results among the PRPs (data not shown). Further, PRP2 was used because it showed clear suppression of HO^•^ production independent of Fenton’s reaction, which is the main result of this study. Indeed, suppression of HO^•^ production, independent of the Fenton’s reaction, was confirmed for all PRPs (data not shown). In histatin research previously reported, an issue was also reported surrounding HO^•^ suppression by Fenton’s reaction using Cu ions. It is, therefore, important to understand the role of Cu ions in the biological system, especially in oral function, and the mechanism of the HO^•^ scavenging effects on salivary protein such as histatin and PRPs. Accordingly, further experiments on these effects should be conducted in the future.

HO^•^ is produced by the biological Harbor Weiss Fenton reaction (Equation 1–3) and is very important in mitigating oxidative stress in diseases, including oral disease ^(2,12,39)^. Since PRPs account for 70% of salivary protein, the evidence that PRPs inhibit HO^•^ produced UV/H_2_O_2_ reaction system independent of Fenton’s reaction (Equation 1, 2), may indicate novel evidence for saliva as part of the oral defense system.

$${Fe}^{3+}\;+\;{O_{2}}^{•-}\to {Fe}^{2+}+O_{2}$$ (1)

$${Fe}^{2+}+H_{2}O_{2}\to {Fe}^{3+}+{OH}^{-}+{HO}^{•}$$ (2)

$${O_{2}}^{•-}+H_{2}O_{2}\to {OH}^{-}+{HO}^{•}+O_{2}$$ (3)

We also investigated the effects of salivary proteins on other HO^•^ generation systems using the UV/H_2_O_2_ reaction system, a well-known Fe^2+^-independent system.^(5,6,34)^ It appears that the HO^•^ scavenging effect of PRP2 is not due to Fe^2^^+^ ion chelation but rather works by directly scavenging HO^•^ (Fig. 2 and 3).

PRP2 did not affect O_2_^•−^ production from the XO and xanthine reaction system (Fig. 4). O_2_^•−^ is known to be an important signaling molecule for various physiological and pathophysiological redox reactions, in various biological systems. PRP2 does not directly eliminate O_2_^•−^ formation, but it does reduce HO^•^ production by the H_2_O_2_/UV irradiation reaction (Fig. 3 and 4). Though PRP2 did not exclude O_2_^•−^, it did display an excellent antioxidant effect against HO^•^ (Fig. 3). In relation to HO^•^, we have found similar results with another salivary protein called histatin.^(34)^ These results suggest that salivary proteins function as an effective defense mechanism against HO^•^ generation which is induced by a disruption in the balance between oxidative stress and antioxidant activity in the oral region.

Acidic PRP has a high affinity for hydroxyapatite minerals on the enamel surface due to the negative charge located at the amino terminus of the molecule.^(43)^ The first 11 amino-terminal residues of acidic PRP contain 8–9 negative charges, including a phosphorylated serine residue at position 8. Also, the first 30 (N-terminal) residues (30rPRP) are involved, including all but three of the negatively charged amino acids of the acidic PRPs. Loss of these N-terminal regions probably eliminates or reduces the normal acidic PRPs function. Therefore, 30rPRP was used to examine which amino acid site of PRP2 is involved in the antioxidant mechanism and whether the antioxidant function of 30rPRP is involved in the physiological function of PRPs. Therefore, to elucidate the antioxidant mechanism of PRP2 structurally, we examined the direct effect of 30 residues of PRP2 (30rPRP, Fig. 1) on ROS generation. 30rPRP did not affect HO^•^ generated by Fenton’s reaction, and O_2_ generation by the X/XO reaction system. However, it suppressed HO^•^ generation by the H_2_O_2_/UV irradiation reaction system (Fig. 5), similar to the results seen with PRP2 (Fig. 3). This suggests that 30rPRP is an important part of the PRP protein structure and plays a significant role in suppressing HO^•^ production. In future experiments, it could be useful to study which specific amino acids within the 30 residues contained in 30rPRP are critical.

A large number of bacteria are found in the oral region; thus, ROS are produced from inflammatory cells on behalf of neutrophils to suppress oral disease-related bacteria. HO^•^ derived from inflammatory cells, O_2_^•−^, acts against bacteria in the oral cavity. In addition, HO^•^ produced by the biological Harbor Weiss Fenton reaction (Equation 1–3) is thought to be involved in oral diseases due to HO^•^ caused by metal ions, such as Cu and Fe ions in the oral cavity. Thus, excessive ROS, such as HO^•^ in chronic inflammation in the oral cavity, is thought to cause oral diseases.^(9–12,42)^ Furthermore, in previous studies, we confirmed the anti-oxidant action of ROS on salivary proteins, such as histatin.^(34)^ Interestingly, we confirmed that the most reactive anti-oxidant activity against HO^•^ was seen in ROS, but no elimination activity against O_2_^•−^. We, therefore, would like to conduct further research on the physiological significance of the antioxidative properties of the amino acid sequence identified in these salivary proteins, such as histatin or PRPs.

In conclusion, we have demonstrated for the first time that PRP directly exhibits antioxidant activity against HO^•^ using the ESR method for our analysis. We also confirmed that 30rPRP is involved in this process. Evidence of the antioxidant effect of PRPs suggests that the antioxidant activity of salivary proteins is important for oral defense against ROS-induced oxidative stress.
